# Supplementary material for: Regulatory role of the N-terminal intrinsically disordered region of the DEAD-box RNA helicase DDX3X in selective RNA recognition
Source: Nat Commun. 2025 Aug 28;16:7762. doi: 10.1038/s41467-025-62806-7 (PMC12394722; doi:10.1038/s41467-025-62806-7)
Supplement: Supplementary file 1 — Supplementary Information [file 41467_2025_62806_MOESM1_ESM.pdf]

Supplementary Information

Regulatory role of the N-terminal intrinsically disordered region of the DEAD-box RNA helicase DDX3X in selective RNA recognition

Yuki Toyama[1,2*] Koh Takeuchi[2*], and Ichio Shimada[1,3*]

1. RIKEN Center for Biosystems Dynamics Research (BDR), 1-7-22 Suehiro-cho, Tsurumi-ku, Yokohama, Kanagawa 230-0045, Japan

2. Graduate School of Pharmaceutical Sciences, The University of Tokyo, 7-3-1 Hongo, Bunkyo-ku, Tokyo 113-0033, Japan

3. Graduate School of Integrated Sciences for Life, Hiroshima University, 1-4-4 Kagamiyama, Higashi-Hiroshima 739-8528, Japan

*Address correspondence to: yuki.toyama@mol.f.u-tokyo.ac.jp, koh-takeuchi@mol.f.u-tokyo.ac.jp, ichio.shimada@riken.jp

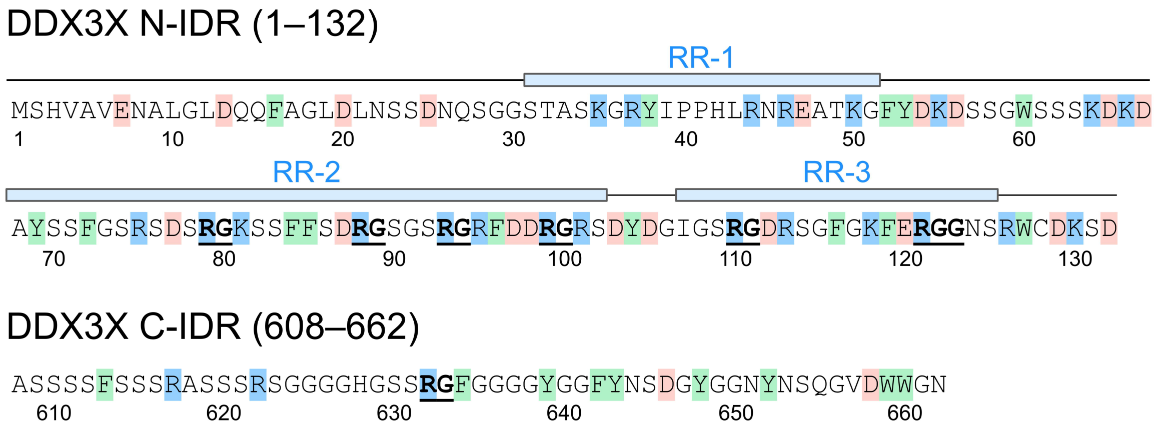

Supplementary Figure 1 The sequences of the N- and C-IDRs of DDX3X. The amino acid sequences of the N-IDR (residues 1–132) and C-IDR (residues 608–662) of human DDX3X are shown. Positively and negatively charged amino acid residues are colored blue and red, respectively, and aromatic amino acid residues are colored green. Three arginine-rich (RR) regions are indicated by blue bars. The RG/RGG motifs are highlighted with underlined bold characters.

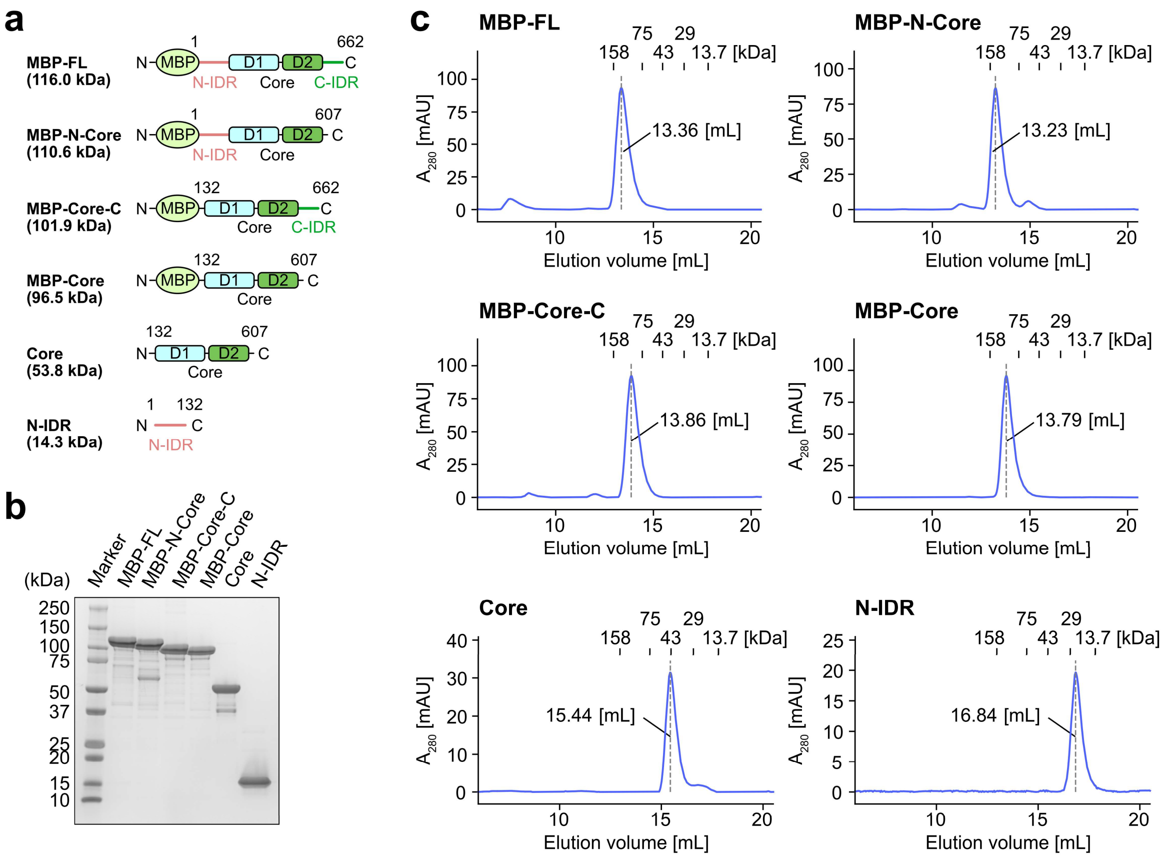

Supplementary Figure 2 Characterization of DDX3X proteins. (a) Domain architecture of the DDX3X variants used in this study. (b) SDS-PAGE analysis of purified proteins. (c) Analytical size-exclusion chromatography of purified proteins. Proteins were separated using a Superdex^{TM} 200 increase column equilibrated with buffer containing 20 mM Tris (pH 8.0), 500 mM NaCl, and 2 mM DTT. For each run, 100 µL of 20 µM protein sample was injected and analyzed at a flow rate of 0.5 mL/min. Molecular weight standards included aldolase (158 kDa), conalbumin (75 kDa), ovalbumin (43 kDa), carbonic anhydrase (29 kDa), and ribonuclease A (13.7 kDa), provided by the Gel Filtration Calibration Kits LMW and HMW (Cytiva 28403841 and 28403842). The *y*-axis is labeled in milli–arbitrary units (mAU). Source data are provided as a Source Data file.

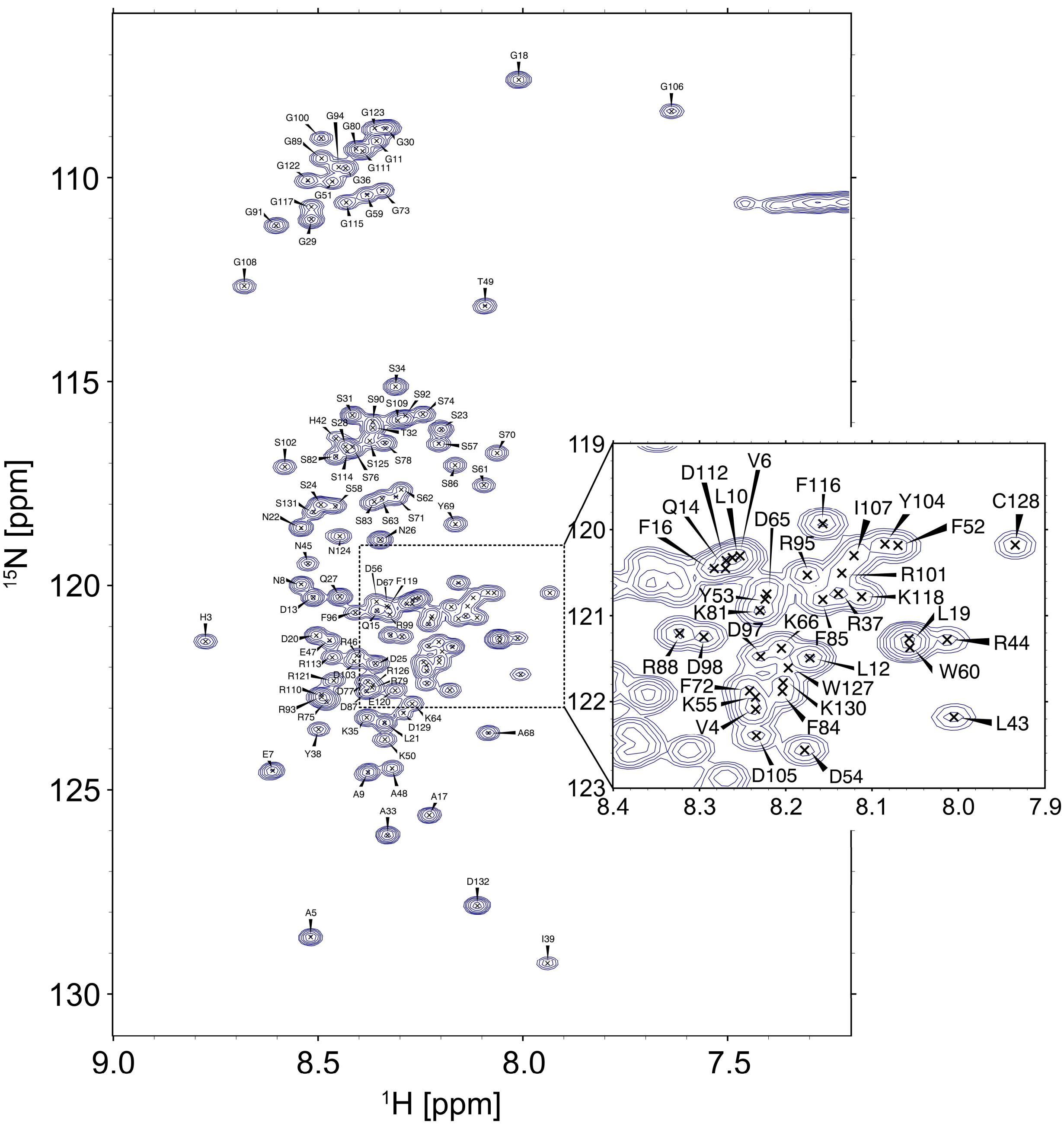

Supplementary Figure 3 Main-chain amide signal assignments of the N-IDR. NMR measurements were performed at 10°C and 600 MHz using a 150 μM [U-^{13}C, ^{15}N]-labeled N-IDR sample. The NMR buffer contained 20 mM potassium phosphate (pH 6.0), 250 mM KCl, 5 mM DTT, and 5% D_2O.

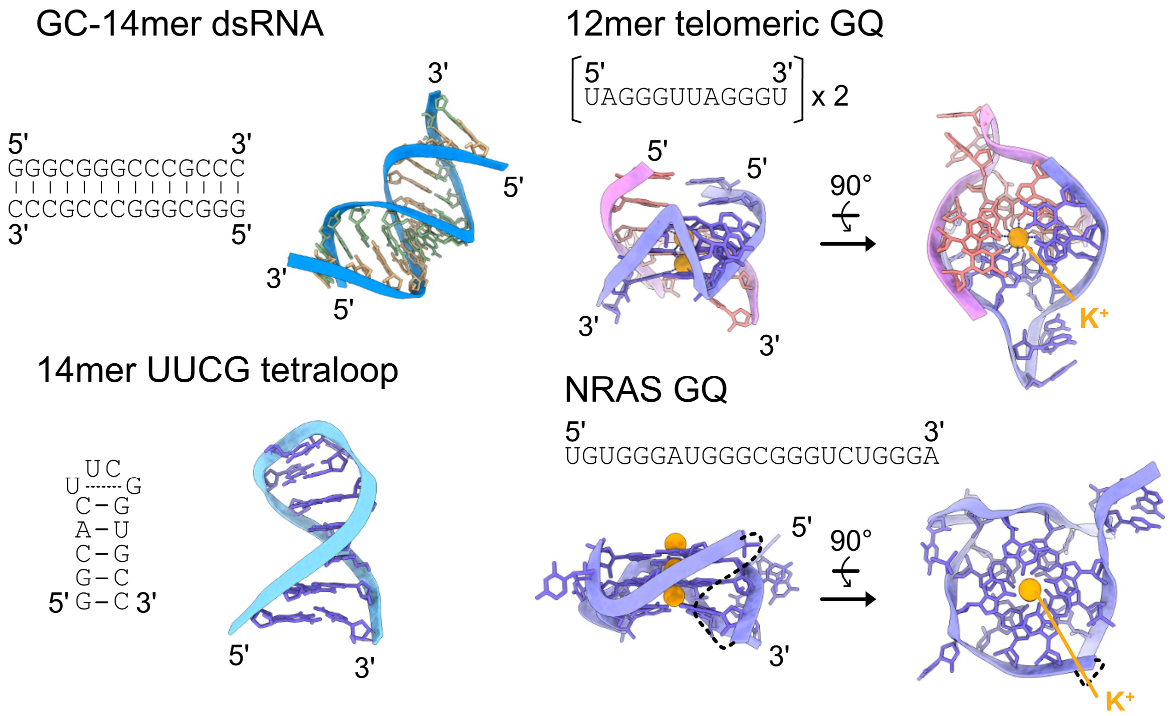

Supplementary Figure 4 The structural models of RNAs used in this study. The structures and sequences of GC-14mer dsRNA, 14mer UUCG tetraloop RNA (PDB ID: 2KOC)[1], 12mer telomeric GQ RNA (PDB: 3IBK)[2], and NRAS GQ RNA (PDB: 7SXP)[3] are shown. The GC-14mer dsRNA structure was modeled using the MacroMoleculeBuilder software (version 3.4)[4] with the crystal structure of 14-bp $[U(UA)_6A]_2$ RNA (PDB ID: 1RNA)[5] as a template. Note that the length and sequence of the NRAS GQ RNA shown in this figure (5′-UGUGGGA<u>U</u>GGGCGGGUCUGGG<u>A</u>-3′) is slightly different from those used in this study (5′-UGUGGGAGGGGCGGGUCUGGGUGC-3′) at underlined positions.

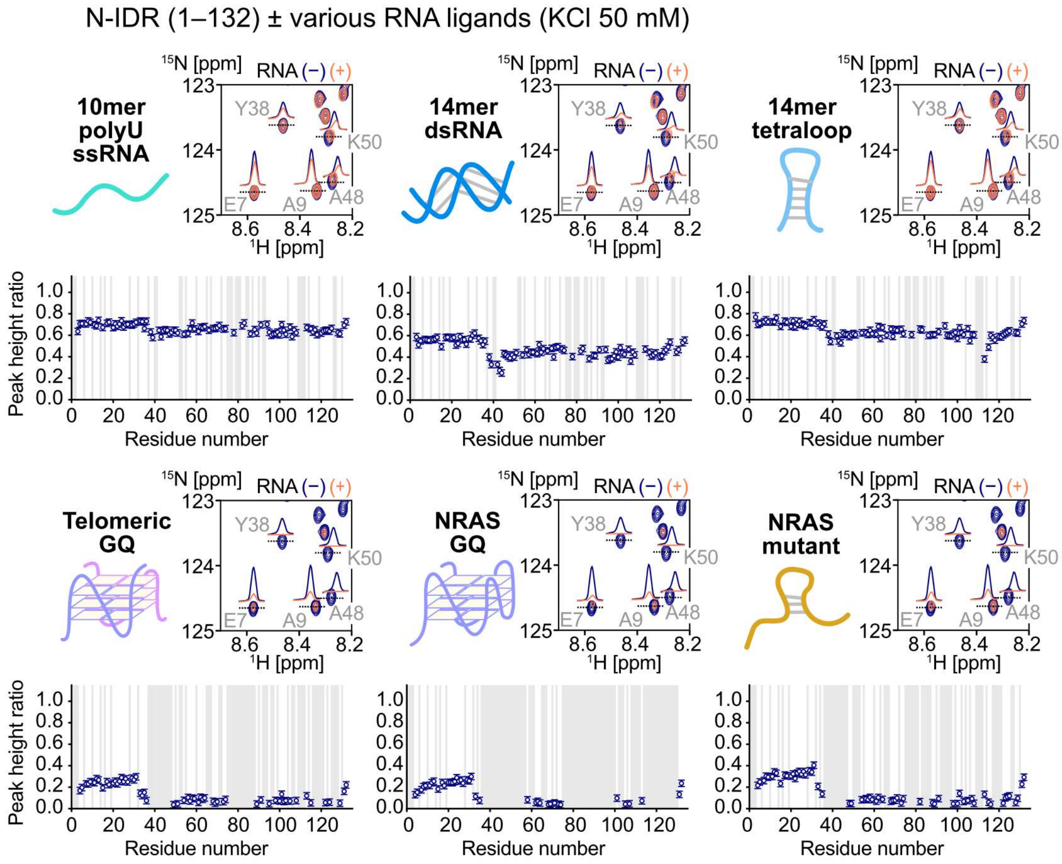

Supplementary Figure 5 NMR characterization of the interaction between the N-IDR and various RNA molecules under low-salt conditions. NMR spectra and peak height ratios of the N-IDR signals obtained with and without each RNA molecule. For each sub-panel, the overlay of ^{15}N-^{1}H HSQC spectra of the [U-^{15}N]-labeled N-IDR in the absence (navy) and presence (orange-red) of RNA, and the plot of peak height ratios are shown. The ratio was calculated by dividing the peak height in the presence of RNA by that in the absence of RNA. Error bars were calculated using the signal-to-noise ratios. Residues that were not analyzed are indicated by gray backgrounds. All NMR measurements were performed at 10°C and 1 GHz in a buffer containing 20 mM potassium phosphate (pH 7.0), 50 mM KCl, 5 mM DTT, 260 units/mL RNAsin® Plus RNAase inhibitor, and 5% D_2O. The protein concentration was 50 μM, and the RNA concentration was 50 μM (for poly-U10 ssRNA, GC-14mer dsRNA, 14mer tetraloop RNA, and telomeric GQ RNA) or 25 μM (for NRAS GQ RNA and NRAS GQ mutant RNA). The 1D slices of the labeled signals are shown in each spectrum. Source data are provided as a Source Data file.

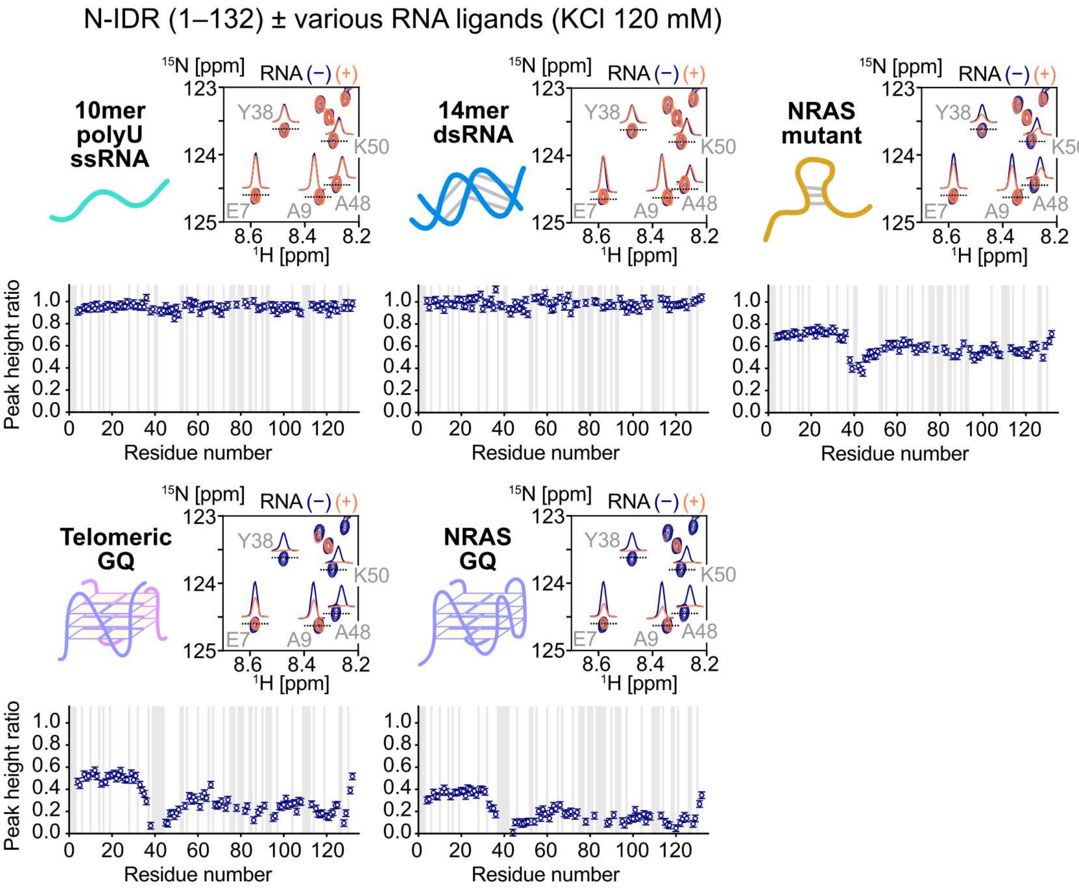

Supplementary Figure 6 NMR characterization of the interaction between the N-IDR and various RNA molecules under physiological salt conditions. The experimental details and plots are the same as in Supplementary Fig. 5, except that all NMR measurements were performed in a buffer containing 20 mM potassium phosphate (pH 7.0), 120 mM KCl, 5 mM DTT, 260 units/mL RNAsin® Plus RNAase inhibitor, and 5% D_2O. Source data are provided as a Source Data file.

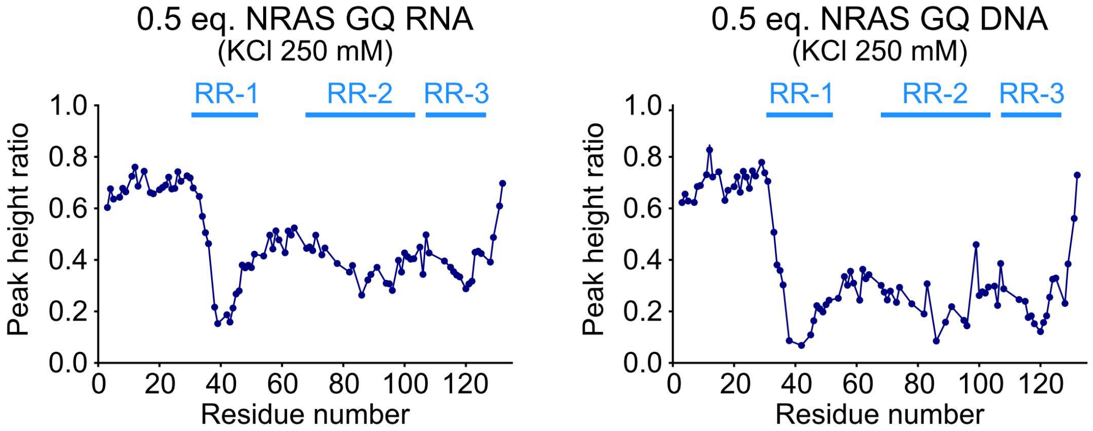

Supplementary Figure 7 NMR characterization of the interaction between the N-IDR and NRAS RNA or DNA. Plots of peak height ratios of the N-IDR signals obtained in the presence and absence of 0.5 eq. NRAS GQ RNA (left) or NRAS GQ DNA (right). The ratio was calculated by dividing the peak height in the presence of RNA or DNA by that in the absence. NMR measurements were performed at 10°C and 1 GHz using [U-^{13}C, ^{15}N]-labeled N-IDR NMR samples containing 20 mM potassium phosphate (pH 7.0), 250 mM KCl, 5 mM DTT, 260 units/mL RNAsin® Plus RNAase inhibitor, and 5% D2O. The protein concentration was 50 μM, and the RNA/DNA concentration was 25 μM. Source data are provided as a Source Data file.

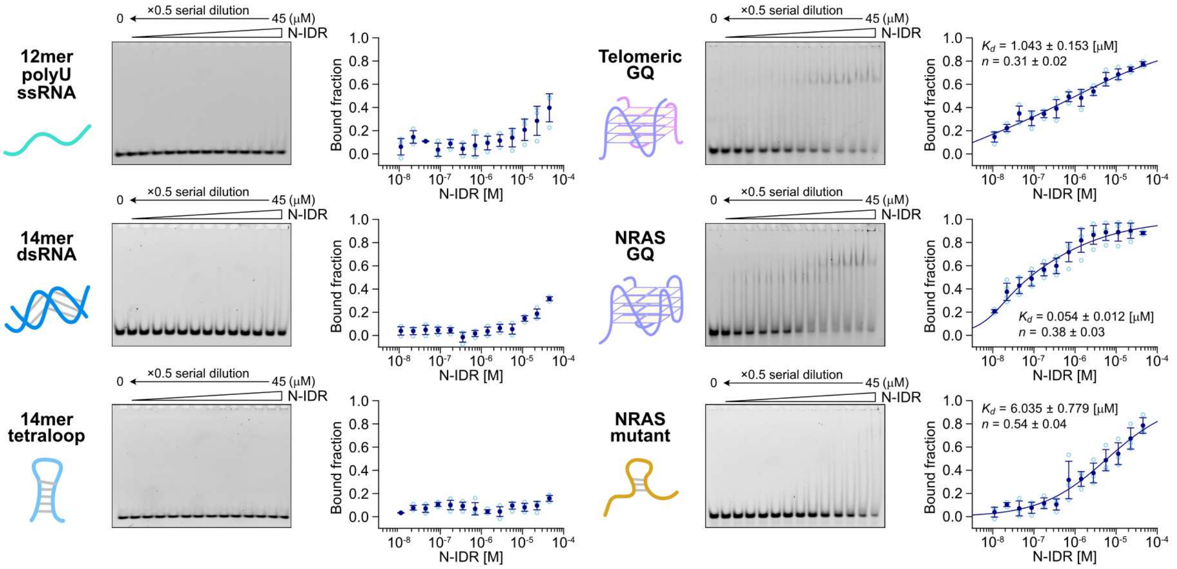

Supplementary Figure 8 EMSA analysis of the interaction between the N-IDR and various RNA ligands. EMSA binding experiments were performed for the N-IDR using poly-U_{12}-FAM ssRNA, GC-14mer-FAM dsRNA, FAM-14mer tetraloop RNA, FAM-telomeric GQ RNA, FAM-NRAS GQ RNA, and FAM-NRAS mutant RNA. Gel images and fitted binding curves used to estimate K_d values are shown. Unlike in the NMR experiment, poly-U_{12} was used instead of poly-U_{10} in EMSA for synthetic reasons. FAM labeling was introduced at the 3′-end for poly-U_{12} ssRNA and GC-14mer dsRNA, and at the 5′-end for all other RNAs. N-IDR protein was titrated from 0 to 45 μM. The bound fraction was estimated from the intensity of the free RNA probe and fitted to a standard Hill-type equation to obtain apparent K_d values. Free and bound probes were separated using polyacrylamide gels: 8% for all RNAs except the 14mer tetraloop, which was run on a 12% gel. All gels were prepared with 0.5× TBE buffer. Error bars represent the standard deviation of four independent measurements for NRAS GQ and the 14mer tetraloop, or three for the others, with center indicating mean values. Source data are provided as a Source Data file.

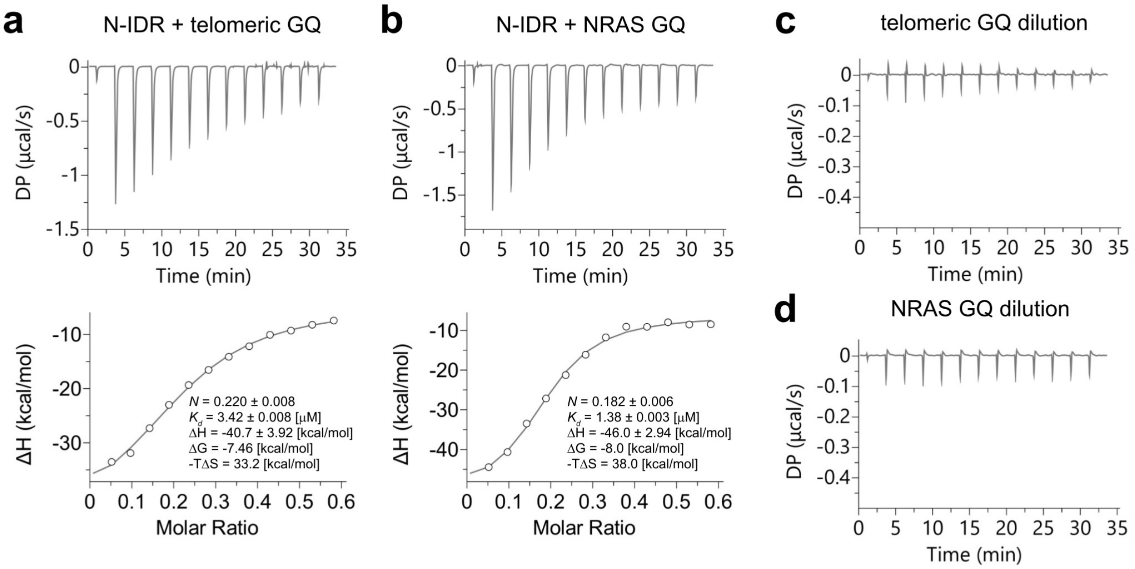

Supplementary Figure 9 ITC experiments for the N-IDR and GQ RNA interactions. (a) ITC isotherm (top) and integrated heats (bottom) for the titration of telomeric GQ RNA (150 μM as a dimer) into a solution of the N-IDR (50 μM). (b) ITC isotherm (top) and integrated heats (bottom) for the titration of NRAS GQ RNA (150 μM as a single strand) into a solution of the N-IDR (50 μM). (c) ITC isotherm for the control experiment involving the dilution of telomeric GQ RNA (150 μM as a dimer) into a solution without the N-IDR. (d) ITC isotherm for the control experiment involving the dilution of NRAS GQ RNA (150 μM as a single strand) into a solution without the N-IDR. The titration data were analyzed using the standard binding model assuming one set of sites, and the fitted parameters are displayed in the insets of (a) and (b). All measurements were performed at 25°C. The experiments were independently repeated at least twice, yielding similar results. During the course of our study, we found that N-IDR molecules can aggregate or coacervate with GQ RNAs at higher concentrations, which are currently under further investigation. We note that heats from such aggregation and/or coacervation of the N-IDR and GQ RNA can also contribute to the measured titration profiles, leading to apparently low *N* values in the fitting process using the simple binding model. Thus, the obtained K_d value should be treated as a rough estimate. Source data are provided as a Source Data file.

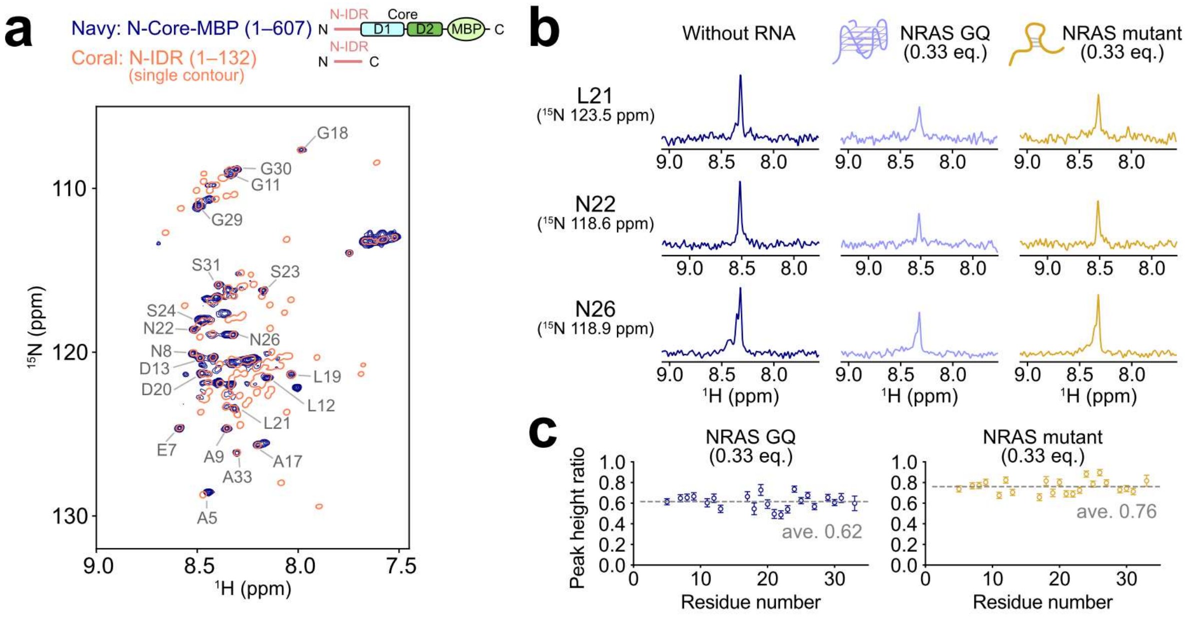

Supplementary Figure 10 NMR characterization of the N-IDR in the full-length protein context. (a) ^{15}N-^{1}H HSQC spectrum of [U-^{15}N]-labeled N-Core-MBP in the absence of RNA (navy, multiple contours). The spectrum of the isolated [U-^{15}N]-labeled N-IDR is overlaid (coral, single contour). (b) 1D slices of the L21, N22, and N26 signals measured in the free state (left), in the presence of 0.33 eq. NRAS GQ RNA (center), or in the presence of 0.33 eq. NRAS mutant RNA (right). (c) Plots of peak height ratios in the presence and absence of 0.33 eq. NRAS GQ RNA (left) or NRAS mutant RNA (right). Error bars were calculated using the signal-to-noise ratios. The average peak height ratio across all residues is indicated by a dashed horizontal line. All NMR measurements were performed at 10°C and 1 GHz in a buffer containing 20 mM potassium phosphate (pH 7.0), 200 mM KCl, 5 mM DTT, 260 units/mL RNAsin® Plus RNAase inhibitor, and 5% D_2O. The protein concentration was 30 μM, and the RNA concentration was 10 μM. Source data are provided as a Source Data file.

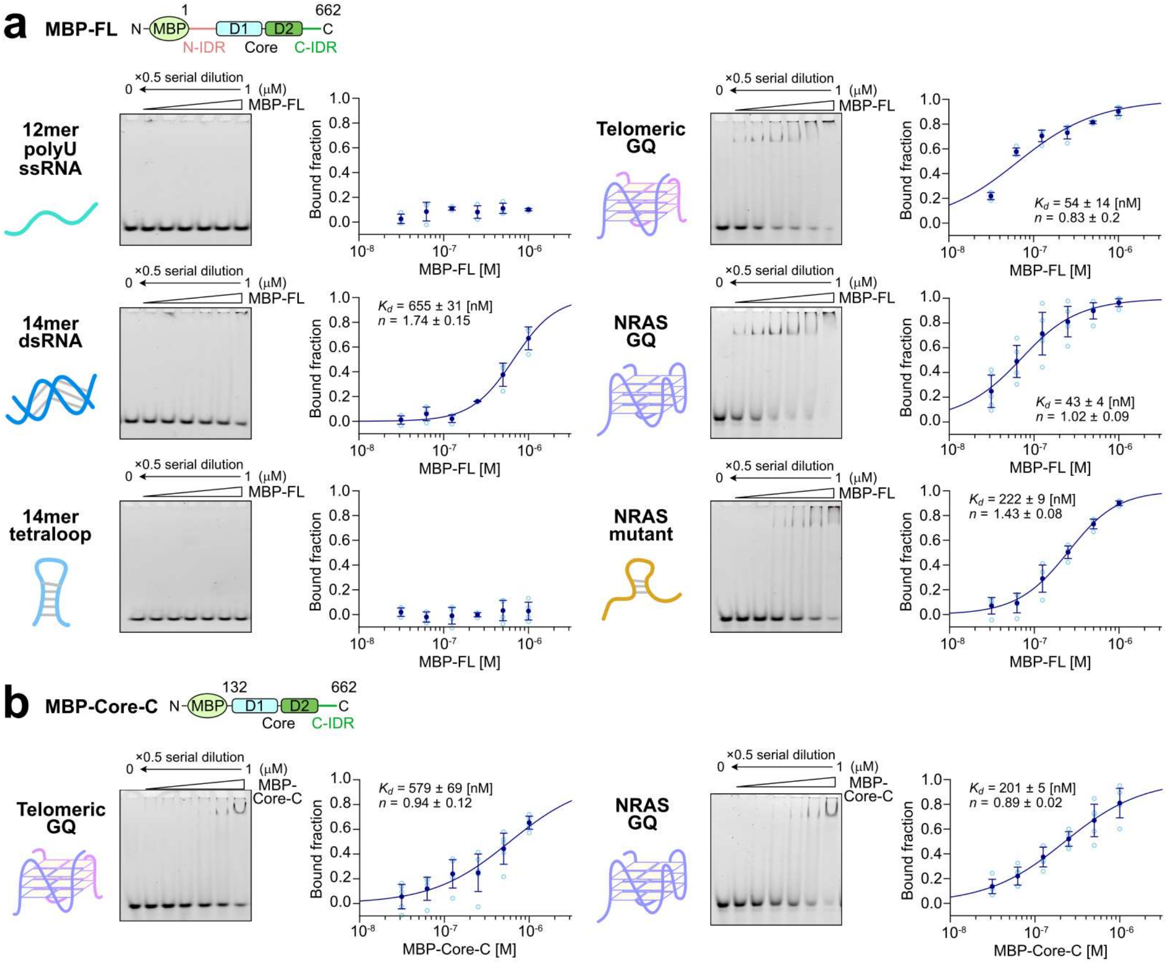

Supplementary Figure 11 EMSA analysis of the interaction between MBP-FL/MBP-Core-C and various RNA ligands. EMSA binding experiments were performed for MBP-FL (a) or MBP-Core-C (b) using poly-U_{12}-FAM ssRNA, GC-14mer-FAM dsRNA, FAM-14mer tetraloop RNA, FAM-telomeric GQ RNA, FAM-NRAS GQ RNA, and FAM-NRAS mutant RNA. Gel images and fitted binding curves used to estimate K_d values are shown. MBP-FL/MBP-Core-C protein was titrated from 0 to 1 μM. The bound fraction was estimated from the intensity of the free RNA probe and fitted to a standard Hill-type equation to obtain apparent K_d values. Free and bound probes were separated using polyacrylamide gels: 8% for all RNAs except the 14mer tetraloop, which was run on a 12% gel. All gels were prepared with 0.5× TBE buffer. Error bars represent the standard deviation of three independent measurements for poly-U_{12} and GC-14mer dsRNA; four for 14mer tetraloop, telomeric GQ RNA (MBP-FL), and NRAS mutant RNA; and five for telomeric GQ RNA (MBP-Core-C) and NRAS GQ RNA (MBP-FL and MBP-Core-C). Centers indicate mean values. Source data are provided as a Source Data file.

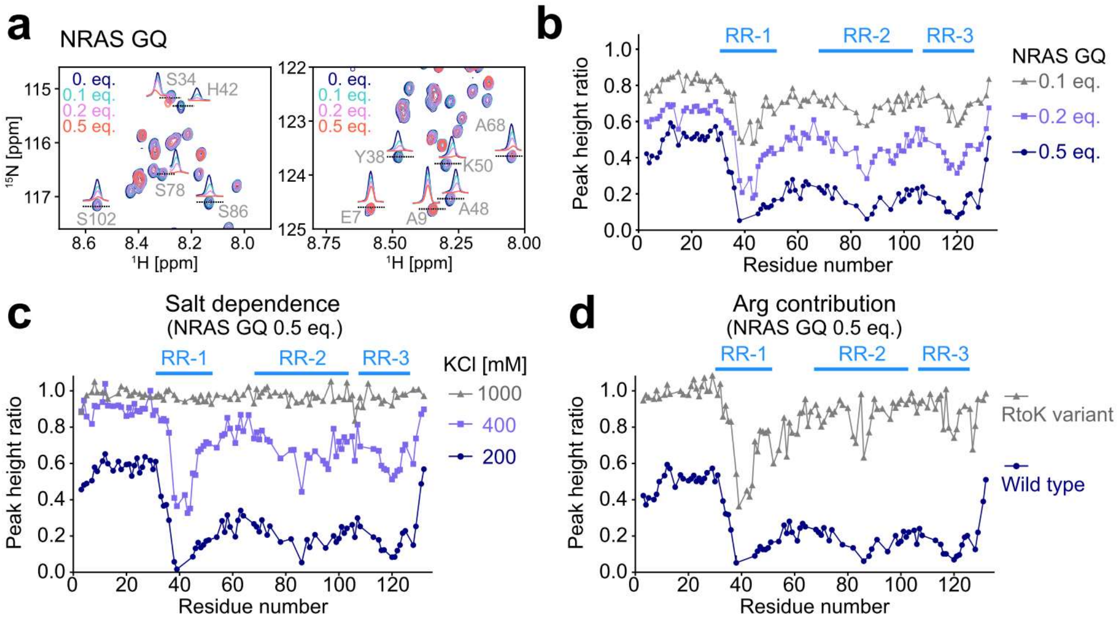

Supplementary Figure 12 NRAS GQ RNA titration experiments. (a) Close-up views of ^{15}N-^{1}H HSQC spectra of the [U-^{15}N]-labeled N-IDR in the presence of varying concentrations of NRAS GQ RNA (navy: 0 equimolar, turquoise: 0.1 equimolar, pink: 0.2 equimolar, and red: 0.5 equimolar). The KCl concentration was 200 mM. The 1D slices of the labeled signals are shown in each spectrum. (b) Plots of the peak height ratios of the N-IDR signals obtained with 0.1 (gray), 0.2 (purple), or 0.5 (navy) equimolar NRAS GQ RNA. (c) Plots of the peak height ratios of the N-IDR signals obtained with 200 mM (navy), 400 mM (purple), or 1 M (gray) KCl. (d) Plots of the peak height ratios of the wild-type (navy) and RtoK variant (gray) N-IDR signals obtained with 200 mM KCl. In panels (c), and (d), the ratio was calculated by dividing the peak height in the presence of 0.5 equimolar (25 µM) NRAS GQ RNA by that in the absence of RNA. All NMR measurements were performed at 10°C and 1 GHz, with a protein concentration of 50 µM. Source data are provided as a Source Data file.

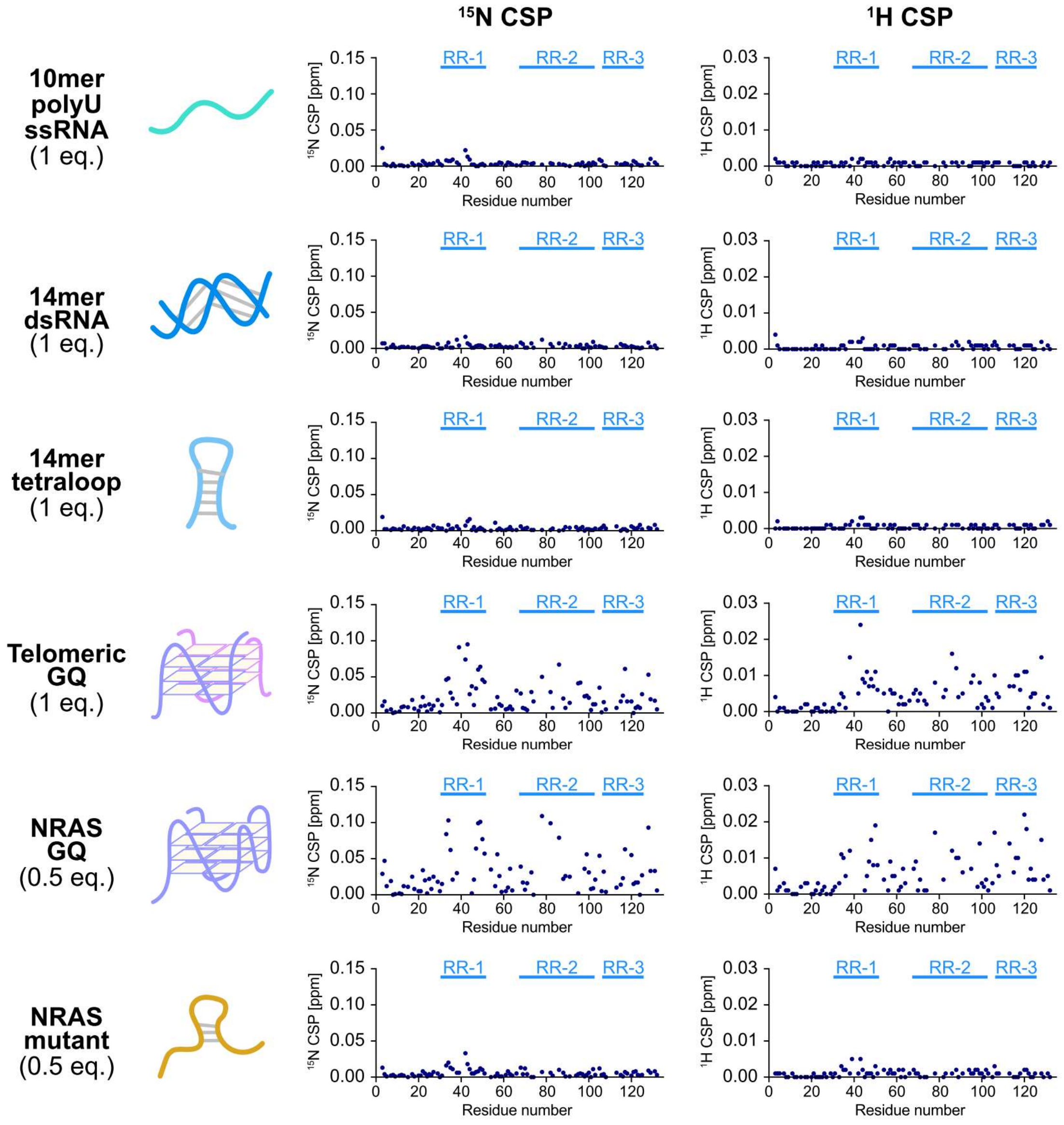

Supplementary Figure 13 Chemical shift perturbations (CSPs) of the N-IDR upon the addition of RNA ligands. Plots of the ^{15}N (left) or ^{1}H (right) absolute chemical shift perturbation values of the [U-^{15}N]-labeled N-IDR observed upon the addition of 1 equimolar (eq.) polyU$_{10}$ ssRNA, 1 eq. GC-14mer dsRNA, 1eq. 14mer tetraloop RNA, 1 eq. telomeric GQ RNA, 0.5 eq. NRAS GQ RNA, and 0.5 eq. NRAS mutant RNA. The three RR clusters in the sequence, defined as RR-1, RR-2, and RR-3, are indicated above each plot. The KCl concentration was 200 mM. All NMR measurements were performed at 10°C and 1 GHz, with a protein concentration of 50 μM. Source data are provided as a Source Data file.

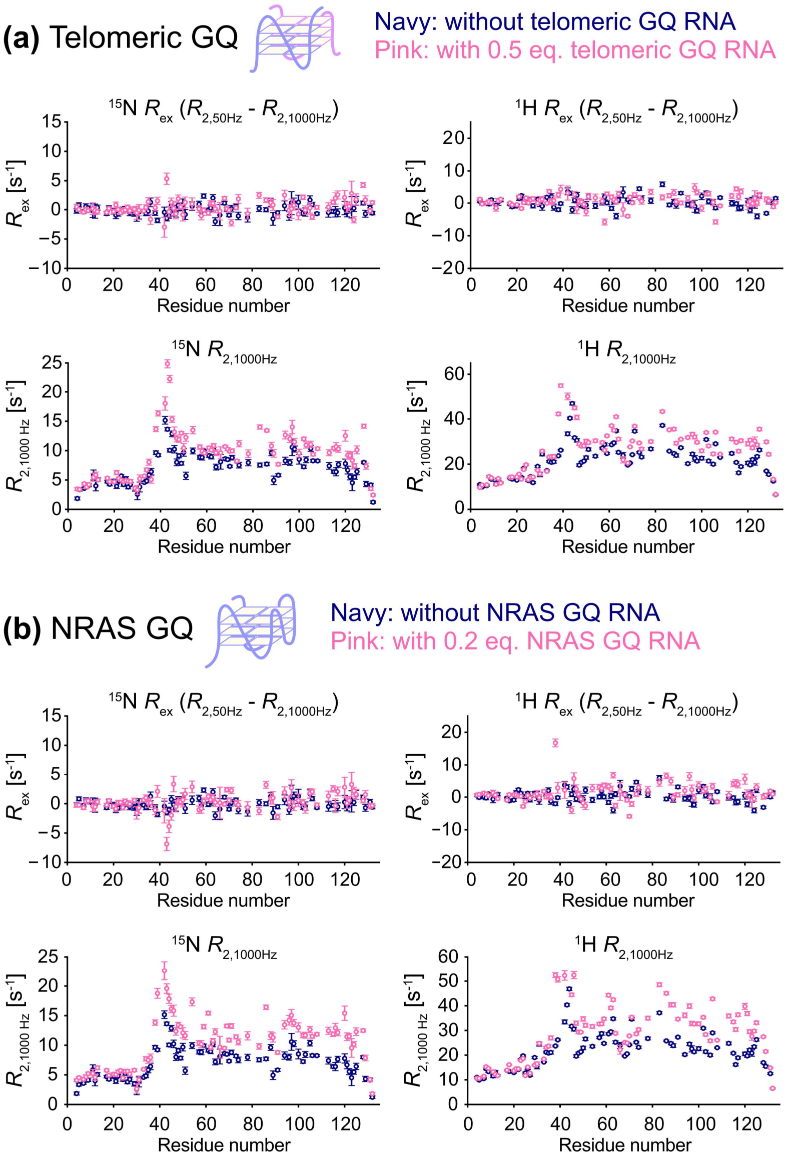

Supplementary Figure 14 ^{15}N and ^{1}H Carr-Purcell-Meiboom-Gill (CPMG) relaxation dispersion analyses of the N-IDR with GQ RNA ligands. Plots of ^{15}N R_{ex} contributions (*top left*), ^{1}H R_{ex} contributions (*top right*), ^{15}N R_2 values measured at a 1,000 Hz CPMG field (*bottom left*), and ^{1}H R_2 values measured at a 1,000 Hz CPMG field (*bottom right*), recorded in the absence (navy) or presence (pink) of GQ RNA. Results with GQ RNA were obtained in the presence of 0.5 eq. telomeric GQ RNA (a) or 0.2 eq. NRAS GQ RNA (b). Error bars were calculated using the signal-to-noise ratios. The KCl concentration was 200 mM. All NMR measurements were performed at 10°C and 1 GHz, with a protein concentration of 50 μM. Source data are provided as a Source Data file.

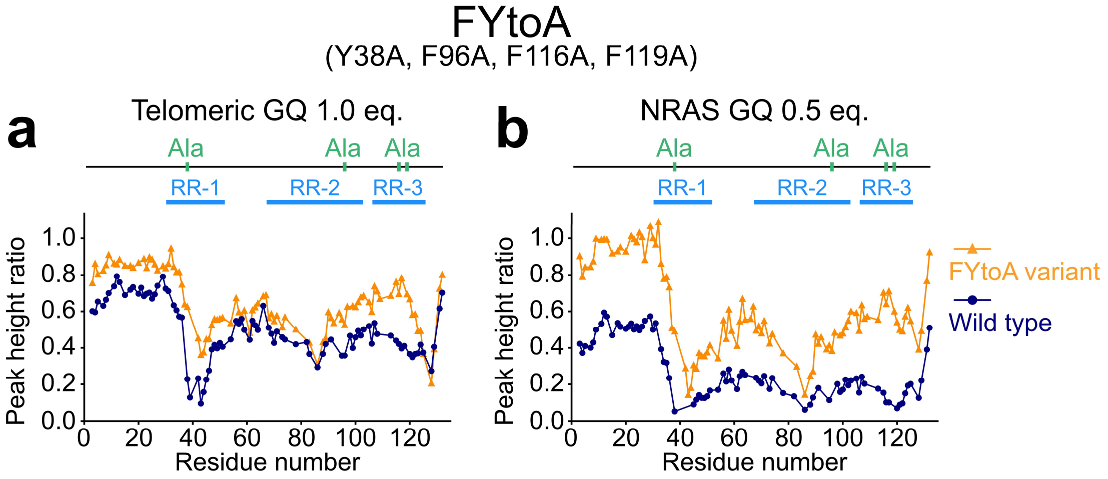

Supplementary Figure 15 GQ RNA titration experiments with the FYtoA variant N-IDR. Plots of the peak height ratios of the wild-type (navy) and FYtoA variant (orange) N-IDR signals obtained with 1.0 equimolar (50 µM) telomeric GQ RNA (a) or 0.5 equimolar (25 µM) NRAS GQ RNA (b) are shown. The ratio was calculated by dividing the peak height in the presence of RNA by that in the absence of RNA. The RR regions are highlighted with blue lines, and the positions of the alanine substitutions are indicated by green lines above the plots. The NMR measurements were performed at 10°C and 1 GHz, with a protein concentration of 50 µM. The NMR buffer contained 20 mM potassium phosphate (pH 7.0), 200 mM KCl, 5 mM DTT, 260 units/mL RNAsin® Plus RNAase inhibitor, and 5% D_2O. Source data are provided as a Source Data file.

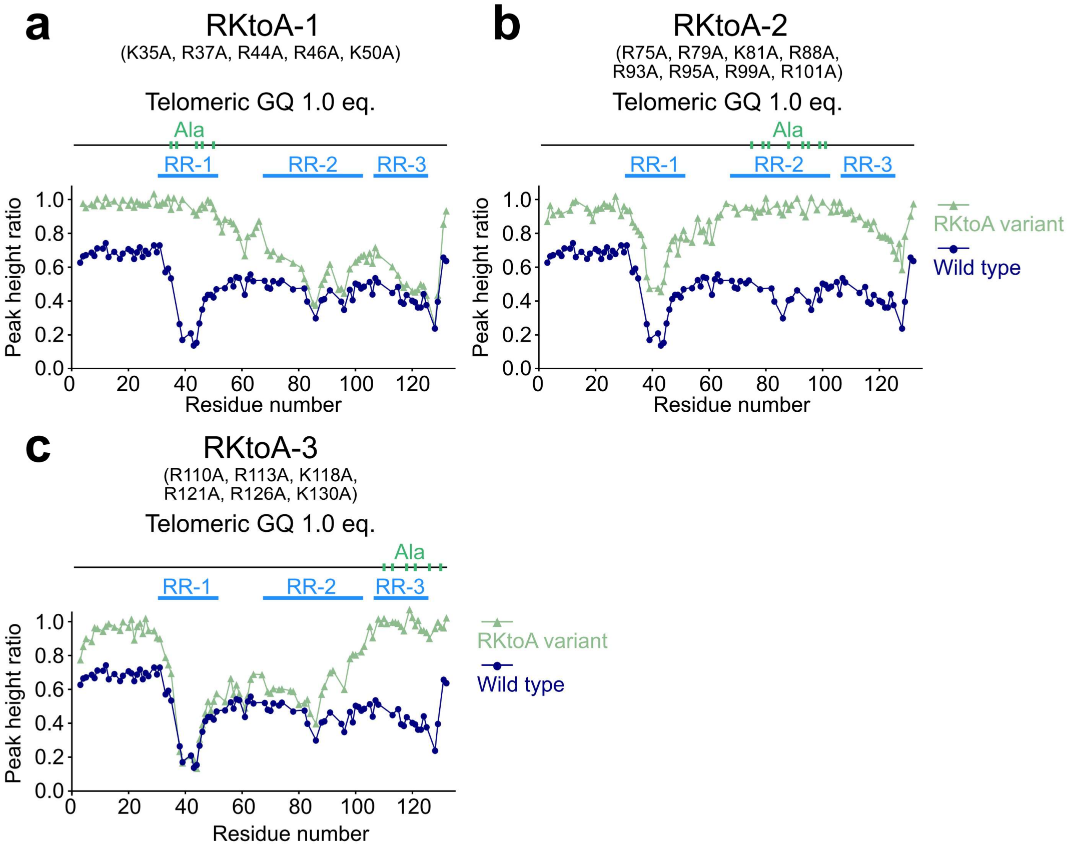

Supplementary Figure 16 Telomeric GQ RNA titration experiments with the RKtoA variant N-IDR. Plots of the peak height ratios of the wild-type (navy) and RKtoA variant (green) N-IDR signals obtained with 1.0 equimolar (50 μM) telomeric GQ RNA are shown. The ratio was calculated by dividing the peak height in the presence of RNA by that in the absence of RNA. The RR regions are highlighted with blue lines, and the positions of the alanine substitutions are indicated by green lines above the plots. The results for RKtoA-1 (a), RKtoA-2 (b), and RKtoA-3 (c) variants are presented. The NMR measurements were performed at 10°C and 1 GHz using [U-^{13}C, ^{15}N]-labeled N-IDR NMR samples containing 20 mM potassium phosphate (pH 7.0), 200 mM KCl, 5 mM DTT, 260 units/mL RNAsin® Plus RNAase inhibitor, and 5% D_2O. Source data are provided as a Source Data file.

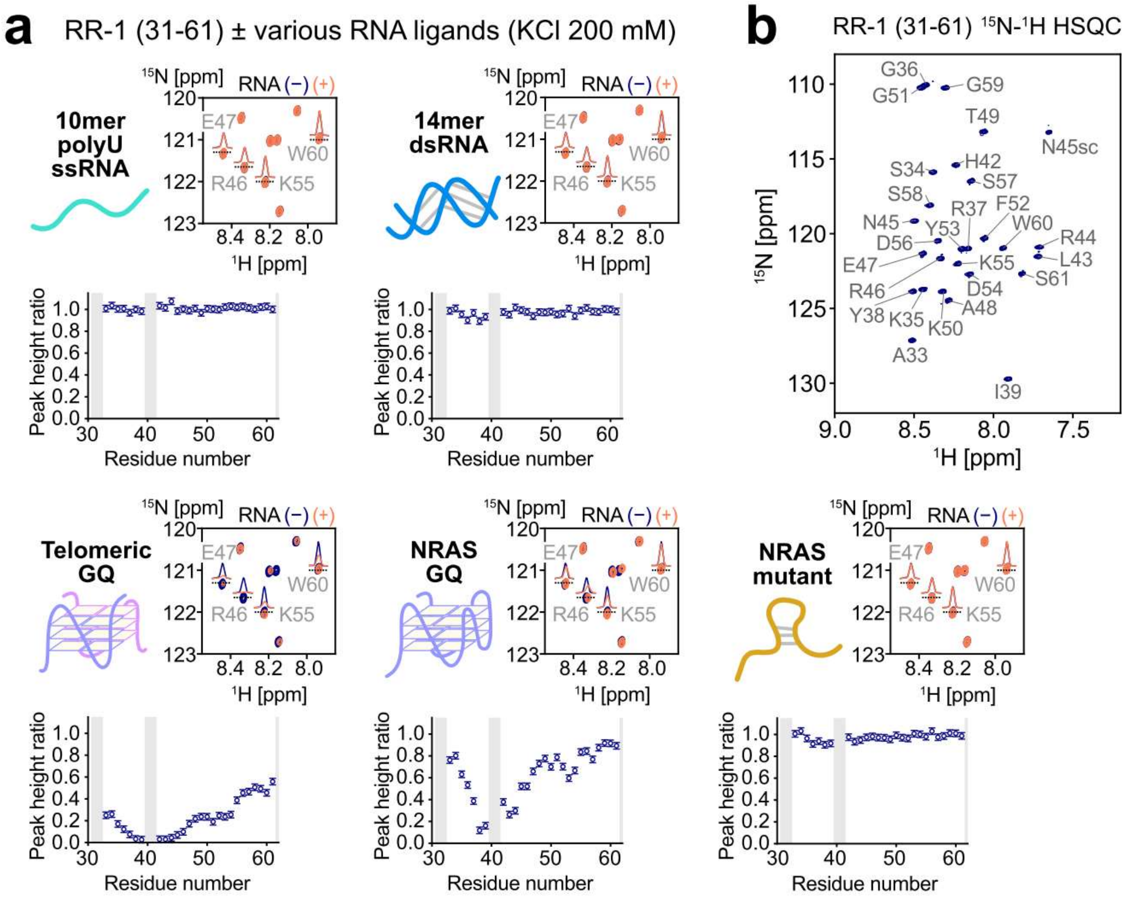

Supplementary Figure 17 NMR characterization of the interaction between the RR-1 fragment and various RNA molecules. (a) NMR spectra and peak height ratios of RR-1 signals obtained with and without each RNA molecule. For each sub-panel, the overlay of ^{15}N-^{1}H HSQC spectra of the [U-^{15}N]-labeled RR-1 in the absence (navy) and presence (orange-red) of RNA, and the plot of peak height ratios are shown. The ratio was calculated by dividing the peak height in the presence of RNA by that in the absence of RNA. Error bars were calculated using the signal-to-noise ratios. Residues that were not analyzed are indicated by gray backgrounds. All NMR measurements were performed at 10°C and 1 GHz in a buffer containing 20 mM potassium phosphate (pH 7.0), 200 mM KCl, 260 units/mL RNAsin® Plus RNAase inhibitor, and 5% D_2O. The protein concentration was 50 μM, and the RNA concentration was 50 μM (for poly-U10 ssRNA, GC-14mer dsRNA, and telomeric GQ RNA) or 25 μM (for NRAS GQ RNA and NRAS GQ mutant RNA). The 1D slices of the labeled signals are shown in each spectrum. (b) Full ^{15}N-^{1}H HSQC spectrum of [U-^{15}N]-labeled RR-1 with signal assignments. Source data are provided as a Source Data file.

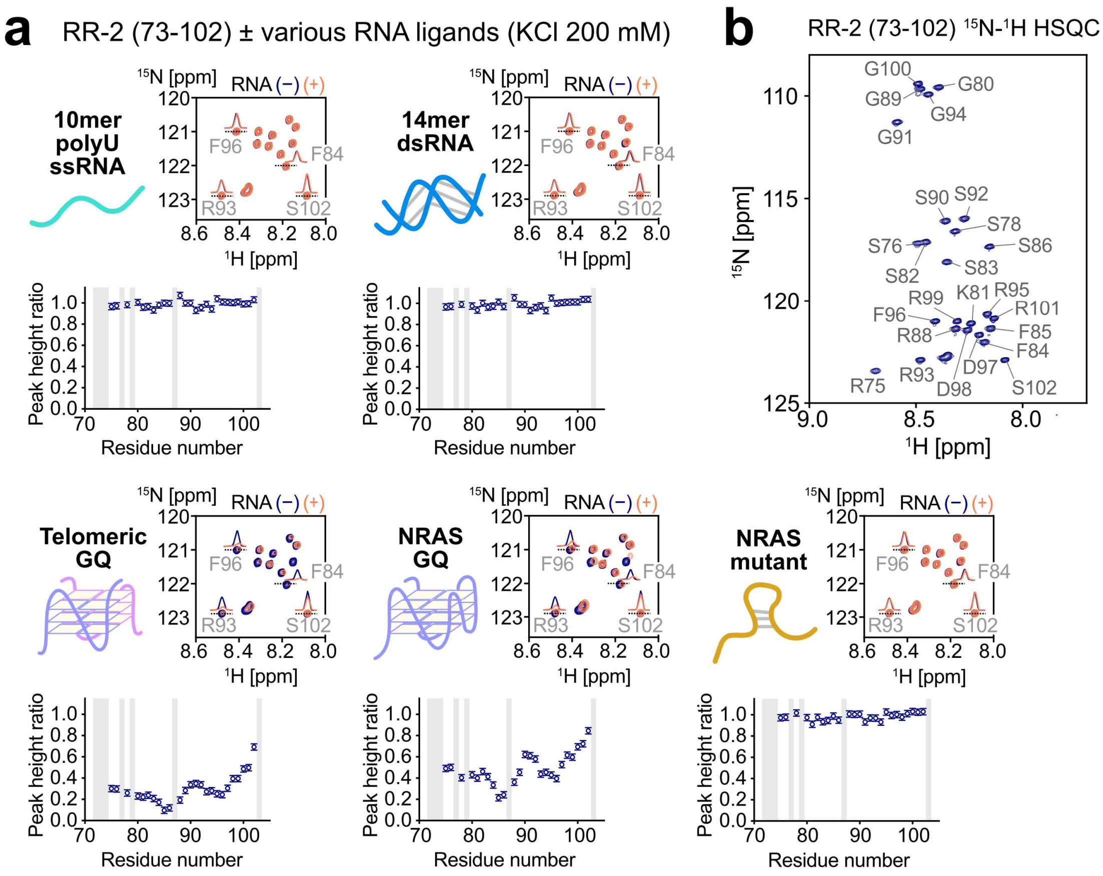

Supplementary Figure 18 NMR characterization of the interaction between the RR-2 fragment and various RNA molecules. The experimental details and plots are the same as in Supplementary Fig. 17.

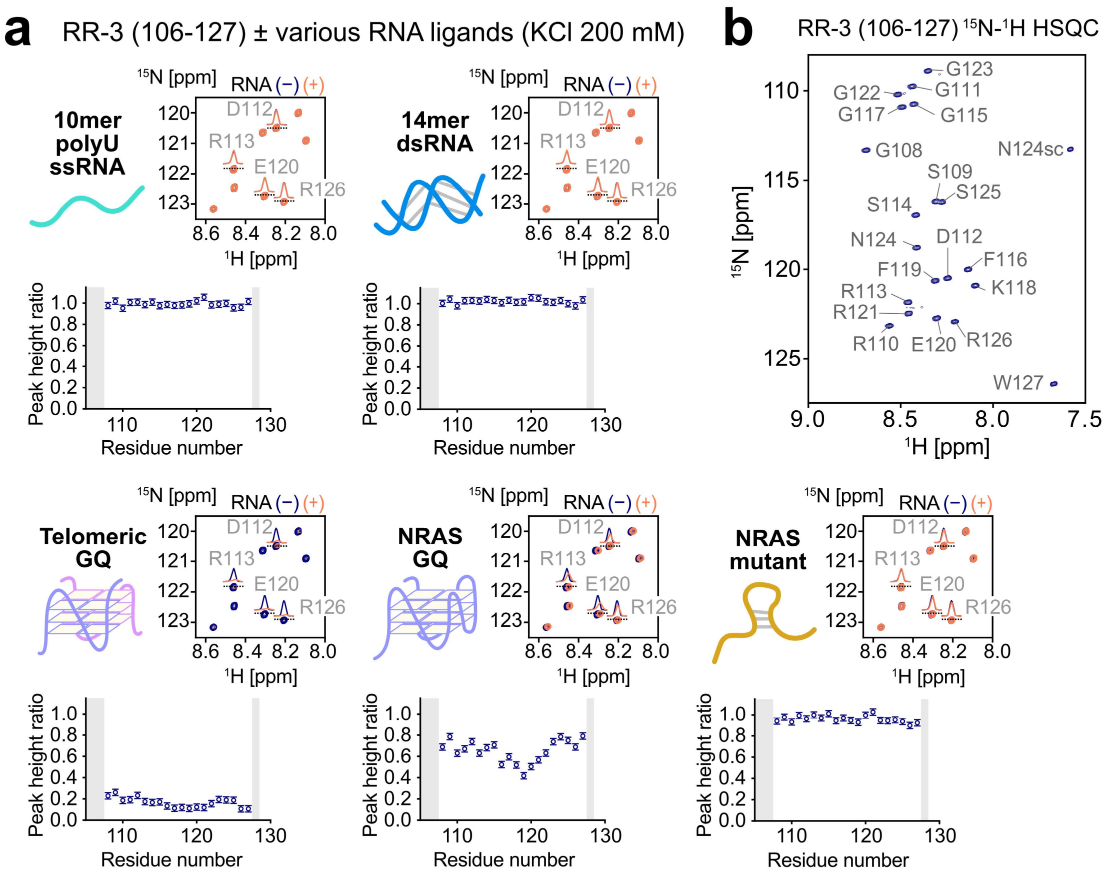

Supplementary Figure 19 NMR characterization of the interaction between the RR-3 fragment and various RNA molecules. The experimental details and plots are the same as in Supplementary Fig. 17.

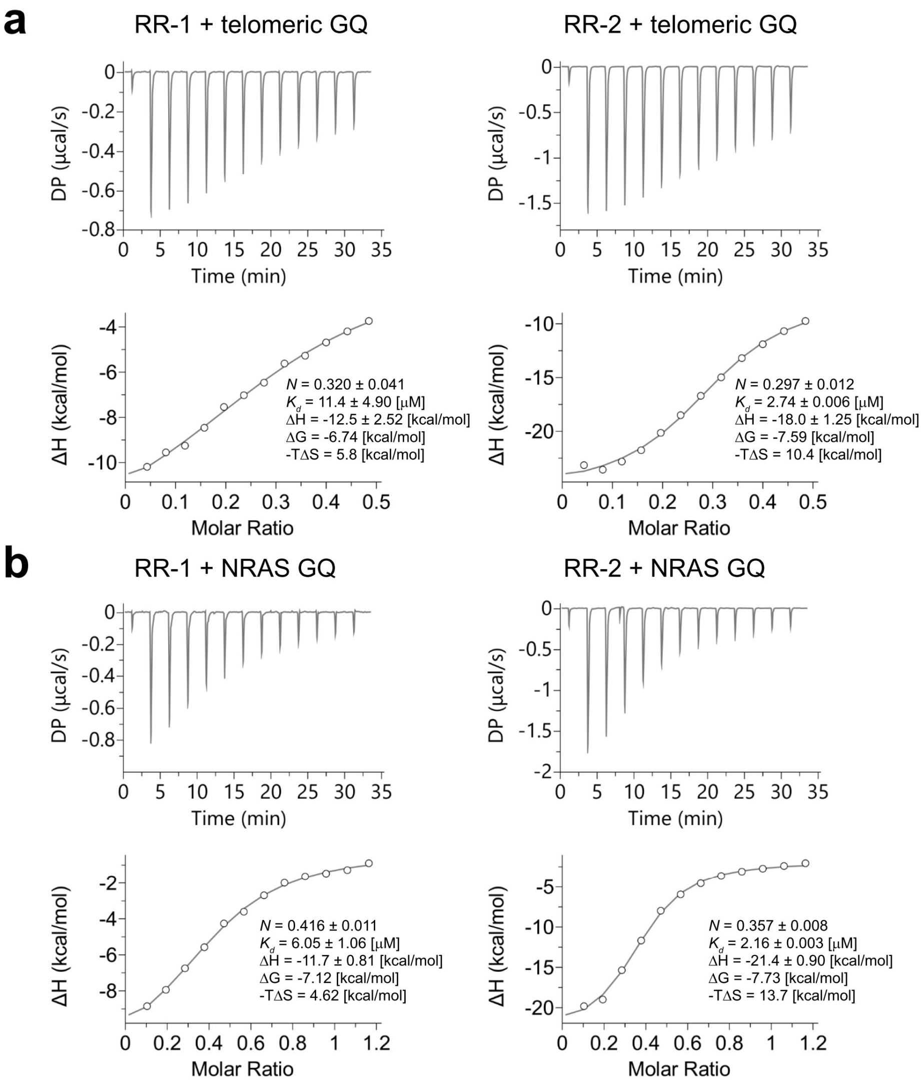

Supplementary Figure 20 ITC experiments for RR-1/RR-2 and GQ RNA interactions. (a) ITC isotherm (top) and integrated heats (bottom) for the titration of telomeric GQ RNA (250 µM as a dimer) into solutions of the RR-1 (left) or RR-2 (right) fragment protein (100 µM). (b) ITC isotherm (top) and integrated heats (bottom) for the titration of NRAS GQ RNA (300 µM as a single strand) into solutions of the RR-1 (left) or RR-2 (right) fragment protein (50 µM). The titration data were analyzed using the standard binding model assuming one set of sites, and the fitted parameters are displayed in the insets. All measurements were performed at 25°C. The experiments were independently repeated at least twice, yielding similar results. Experiments on RR-3 were not included due to poor reproducibility, likely due to a tendency to form small amounts of aggregates. Source data are provided as a Source Data file.

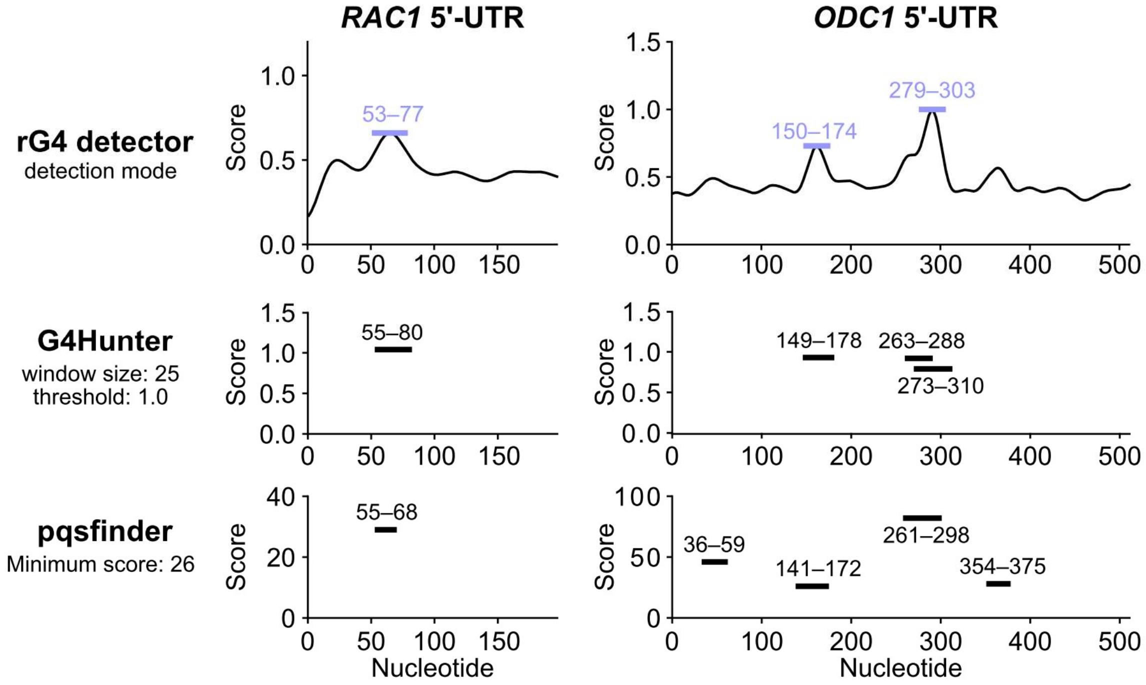

Supplementary Figure 21 GQ propensity of *RAC1* and *ODC1* 5′-UTR calculated using the rG4 detector, G4Hunter, and pqsfinder software. Plots of the prediction scores for GQ propensity of *RAC1* (left) and *ODC1* (right) 5′-UTR sequences obtained using the rG4 detector[6] (top), G4Hunter[7] (middle), and pqsfinder[8] (bottom) software. In the analyses using the rG4 detector software, a prediction score is assigned for each nucleotide. In the analyses using the G4Hunter and pqsfinder software, putative GQ-forming segments are identified with a prediction score. In the predictions using G4Hunter, the minimum length and threshold were set to 25 and 1.0, respectively. In the predictions using pqsfinder, the minimum score was set to 26. The score values are indicated on the *y*-axis. Source data are provided as a Source Data file.

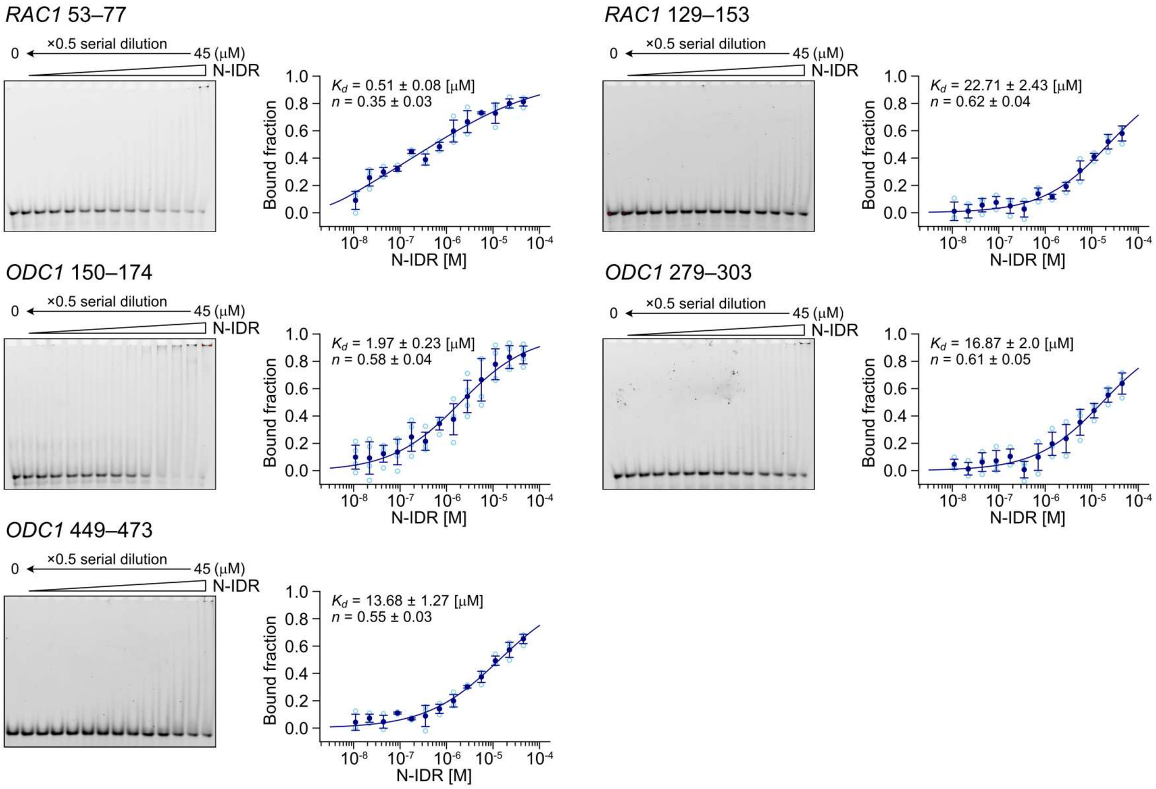

Supplementary Figure 22 EMSA analysis of the interaction between the N-IDR and mRNA fragments. EMSA binding experiments were performed using the N-IDR and the following FAM-labeled mRNA fragments: *RAC1* 53–77, *RAC1* 129–153, *ODC1* 150–174, *ODC1* 279–303, and *ODC1* 449–473. Gel images and fitted binding curves used to estimate K_d values are shown. FAM labeling was introduced at the 5′-end for all mRNA fragments. N-IDR protein was titrated from 0 to 45 μM. The bound fraction was estimated from the intensity of the free RNA probe and fitted to a standard Hill-type equation to obtain apparent K_d values. Samples were separated using 8% polyacrylamide gels prepared with 0.5× TBE buffer. Error bars represent the standard deviation of four independent measurements for *ODC1* 150–174, or three for the others, with center indicating mean values. Source data are provided as a Source Data file.

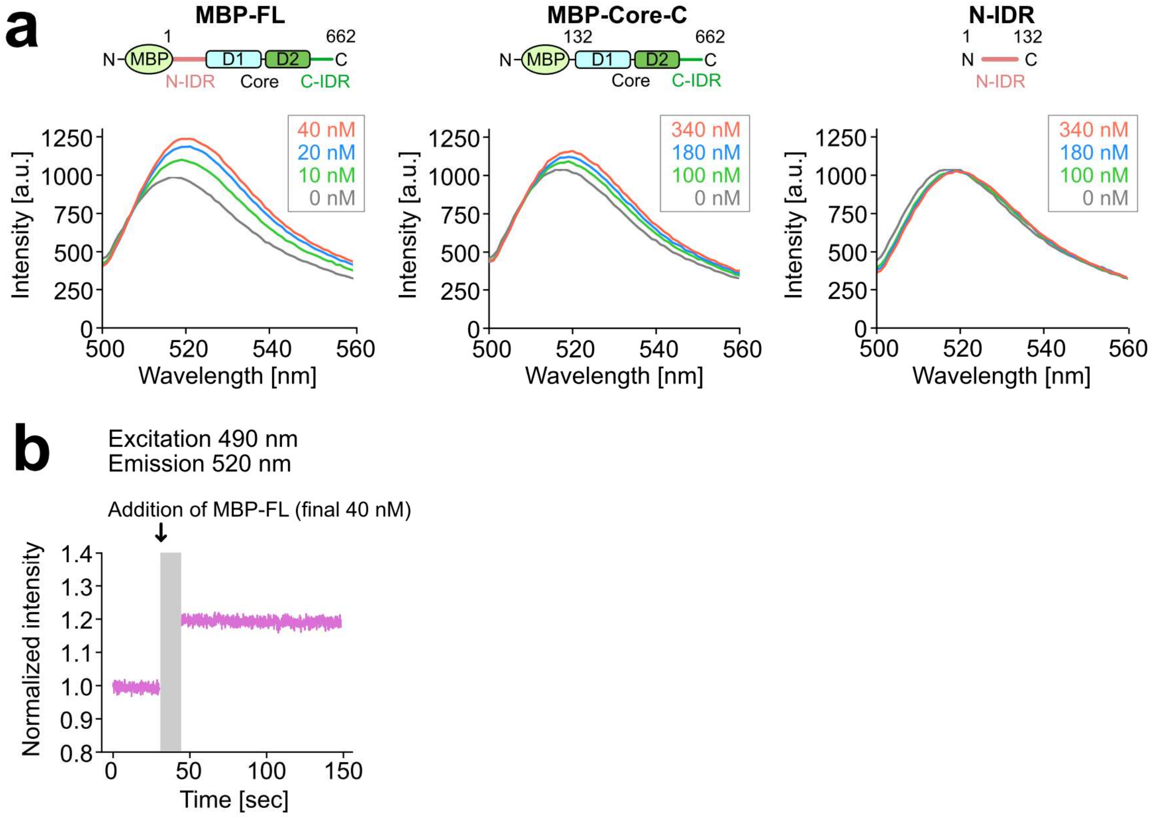

Supplementary Figure 23 GQ-unfolding activity measured via fluorescence quenching. (a) Representative fluorescence emission spectra of FAM/BHQ1-labeled NRAS GQ recorded in the absence (gray line) and presence (colored lines) of varying concentrations of MBP-FL (left), MBP-Core-C (middle), and N-IDR (right) proteins. Protein concentrations ranged from 0 to 40 nM for MBP-FL and from 0 to 340 nM for MBP-Core-C and N-IDR. The excitation wavelength was set to 490 nm. (b) Time-course analysis of GQ-unfolding upon addition of 40 nM MBP-FL to FAM/BHQ1-labeled NRAS GQ. At the time point indicated by the arrow, 100 μL of MBP-FL solution was rapidly added to a 3 mL sample containing 20 nM FAM/BHQ1-labeled NRAS GQ. The gray background indicates the dead time during MBP-FL addition. The excitation and emission wavelengths were set to 490 nm and 520 nm, respectively. The analyses were independently repeated twice with consistent results. Source data are provided as a Source Data file.

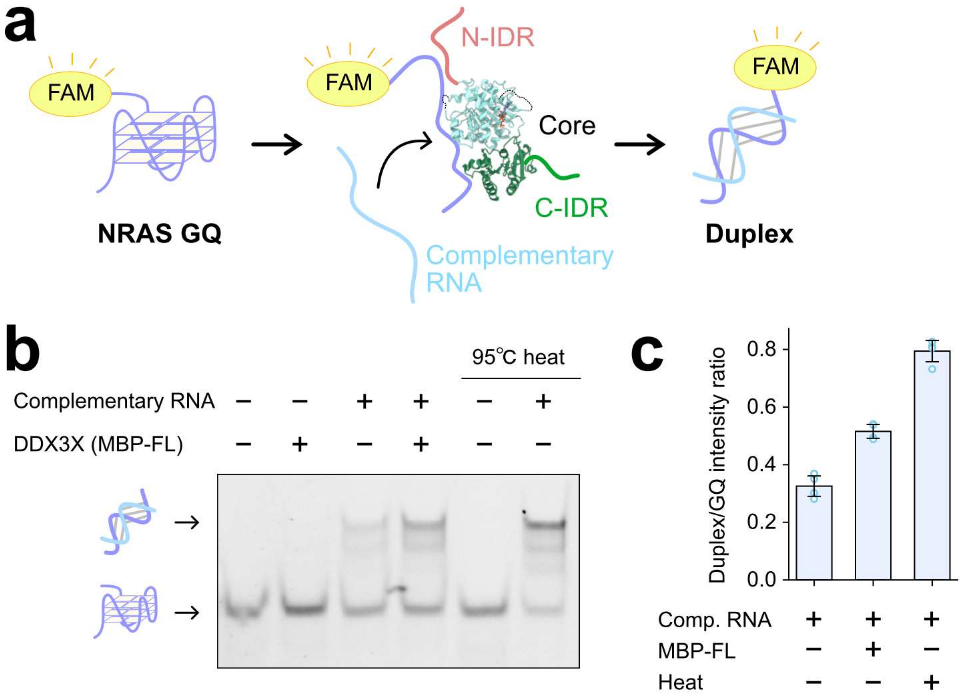

Supplementary Figure 24 GQ-unfolding assay monitored by hybridization to a complementary RNA strand. (a) Schematic representation of the NRAS GQ-unfolding assay, in which GQ-unfolding is detected by hybridization to a complementary RNA strand. (b) Native gel analysis of reaction mixtures containing 50 nM FAM-labeled NRAS GQ RNA, with or without 100 nM complementary RNA, and with or without 100 nM DDX3X (MBP-FL). The two rightmost lanes show samples incubated at 95°C for 3 minutes prior to electrophoresis. Samples were separated using 20% polyacrylamide gels prepared with Tris-Glycine buffer. (c) Plots of the intensity ratio between the duplex and NRAS GQ bands for lanes containing 100 nM complementary strand. Error bars represent the standard deviation of four independent measurements, with center indicating mean values. Source data are provided as a Source Data file.

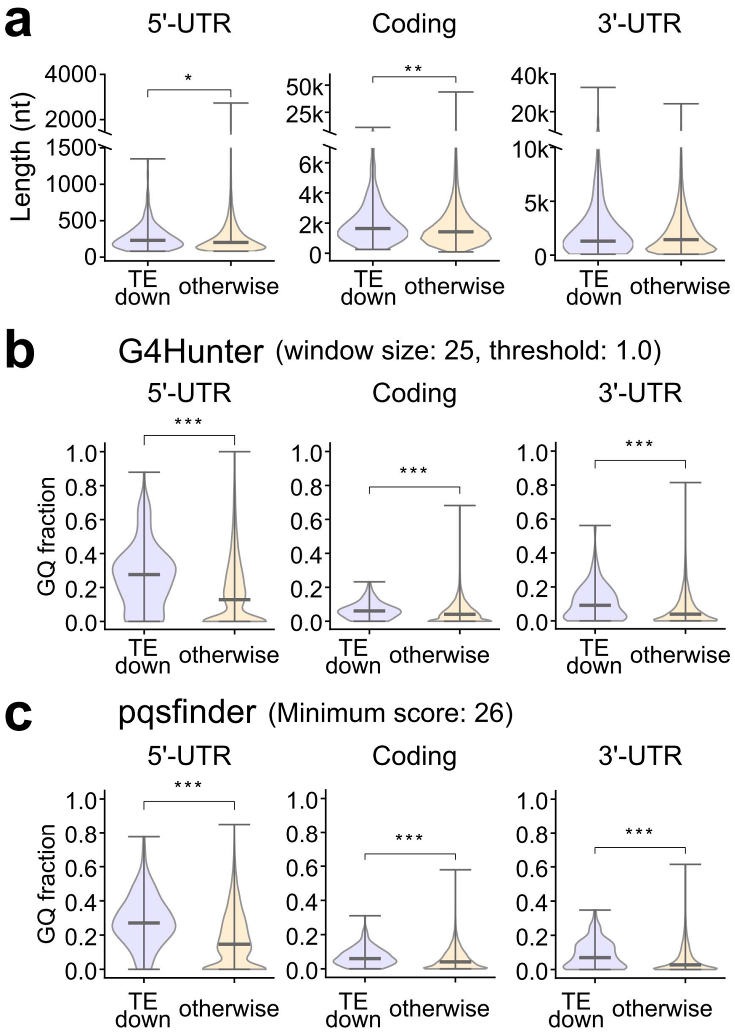

Supplementary Figure 25 Plots of the length and GQ fraction of DDX3X's targets. (a) Violin plots comparing the lengths of each transcript. (b) Violin plots comparing the distributions of the predicted GQ fractions obtained using the G4Hunter[7] software. (c) Violin plots comparing the distributions of the predicted GQ fractions obtained using the pqsfinder[8] software. In each plot, the distributions were compared between two groups, DDX3X targets (TE down; 208 transcripts) and non-targets (otherwise; 8,783 transcripts). Three regions, 5′-UTR (left), protein coding (center), and 3′-UTR (right), were analyzed separately. The horizontal bars in the violin plots indicate the median values, while the top and bottom error bars represent the maximum and minimum values, respectively. Statistical significance between the two groups was evaluated using the two-sided Mann-Whitney U test (***: $p < 0.001$, **: $0.001 < p < 0.01$, and *: $0.01 < p < 0.05$). All relevant data and exact p values are included in the Source Data file.

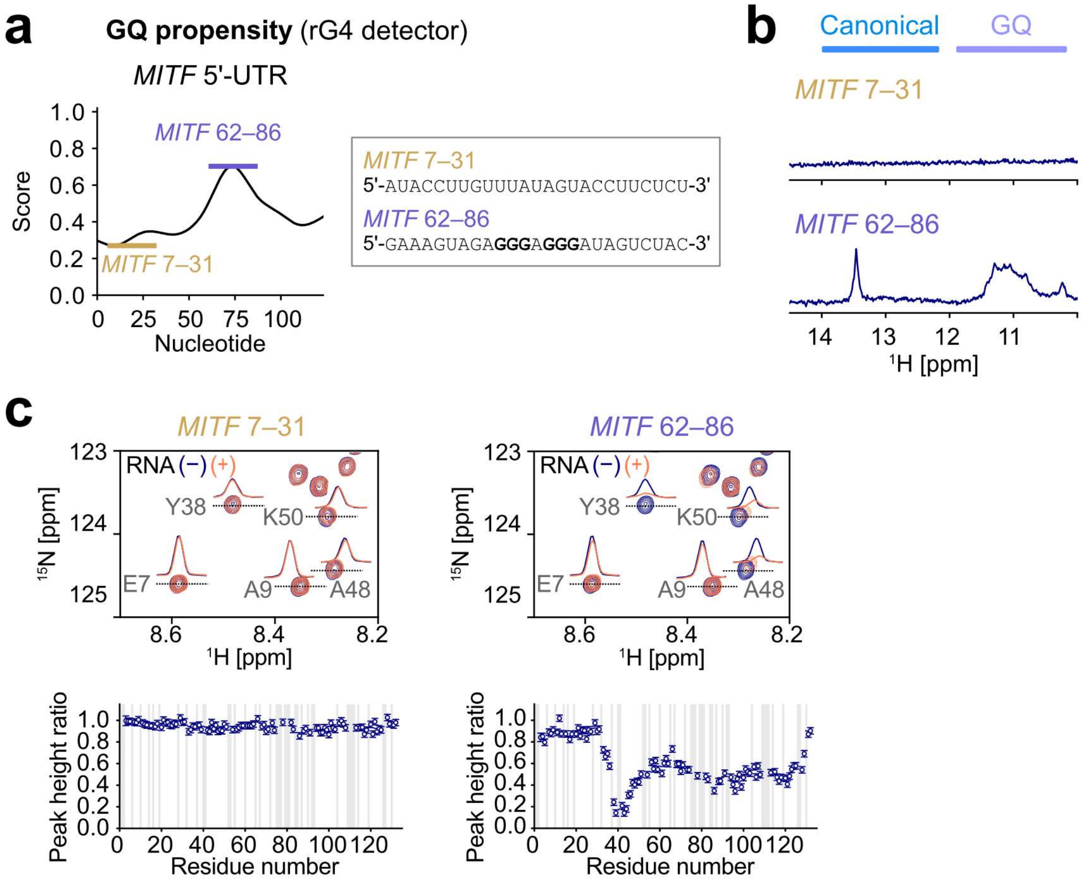

Supplementary Figure 26 GQ propensity of *MITF* and its interaction with the N-IDR. (a) Plots of the prediction scores for GQ propensity of the *MITF* 5′-UTR sequence obtained using the rG4 detector software[6]. In the inset, the sequences of the segment with high prediction scores (*MITF* 62–86, purple) and the negative control (*MITF* 7–31, gold) are highlighted. (b) The imino ^{1}H 1D NMR spectra of the *MITF* 7–31 (top) and *MITF* 62–86 fragments. (c) NMR spectra and peak height ratios of the N-IDR signals obtained with and without each RNA fragment in the presence of 200 mM KCl. In the top row, overlays of ^{15}N-^{1}H HSQC spectra of the [U-^{15}N]-labeled N-IDR in the absence (navy) and presence (orange-red) of RNA are shown. The 1D slices of the labeled signals are shown in each spectrum. In the bottom row, plots of peak height ratios are shown. The ratio was calculated by dividing the peak height in the presence of 0.5 equimolar RNA by that in the absence of RNA. Error bars were calculated using the signal-to-noise ratios. Residues that were not analyzed are indicated with gray backgrounds. All NMR measurements were performed at 10°C and 1 GHz, and protein and RNA concentrations were 50 µM and 25 µM, respectively. Source data are provided as a Source Data file.

Supplementary References

1. Nožinović, S., Fürtig, B., Jonker, H. R., Richter, C. & Schwalbe, H. High-resolution NMR structure of an RNA model system: the 14-mer cUUCGg tetraloop hairpin RNA. *Nucleic Acids Res.* **38**, 683–694 (2009).
2. Collie, G. W., Haider, S. M., Neidle, S. & Parkinson, G. N. A crystallographic and modelling study of a human telomeric RNA (TERRA) quadruplex. *Nucleic Acids Res.* **38**, 5569–5580 (2010).
3. Balaratnam, S. *et al.* Investigating the NRAS 5' UTR as a target for small molecules. *Cell Chem. Biol.* **30**, 643-657.e8 (2023).
4. Flores, S. C., Sherman, M. A., Bruns, C. M., Eastman, P. & Altman, R. B. Fast flexible modeling of RNA structure using internal coordinates. *IEEE/ACM Trans. Comput. Biol. Bioinform.* **8**, 1247–1257 (2011).
5. Dock-Bregeon, A. C. *et al.* Crystallographic structure of an RNA helix: [U(UA)6A]2. *J. Mol. Biol.* **209**, 459–474 (1989).
6. Turner, M. *et al.* rG4detector, a novel RNA G-quadruplex predictor, uncovers their impact on stress granule formation. *Nucleic Acids Res.* **50**, 11426–11441 (2022).
7. Bedrat, A., Lacroix, L. & Mergny, J.-L. Re-evaluation of G-quadruplex propensity with G4Hunter. *Nucleic Acids Res.* **44**, 1746–1759 (2016).
8. Hon, J., Martínek, T., Zendulka, J. & Lexa, M. pqsfinder: an exhaustive and imperfection-tolerant search tool for potential quadruplex-forming sequences in R. *Bioinformatics* **33**, 3373–3379 (2017).
