# Supplementary material for: Regulatory role of the N-terminal intrinsically disordered region of the DEAD-box RNA helicase DDX3X in selective RNA recognition
Source: Nat Commun. 2025 Aug 28;16:7762. doi: 10.1038/s41467-025-62806-7 (PMC12394722; doi:10.1038/s41467-025-62806-7)
Supplement: Supplementary file 2 — Description of Additional Supplementary Files [file 41467_2025_62806_MOESM2_ESM.pdf]

Descriptions of Additional Supplementary Files

Supplementary Data 1: DNA sequences of DDX3X constructs and primers.

Supplementary Data 2: RNA sequences used in this study.
