# Supplementary material for: Regulatory role of the N-terminal intrinsically disordered region of the DEAD-box RNA helicase DDX3X in selective RNA recognition
Source: Nat Commun. 2025 Aug 28;16:7762. doi: 10.1038/s41467-025-62806-7 (PMC12394722; doi:10.1038/s41467-025-62806-7)
Supplement: Supplementary file 5 — Reporting Summary [file 41467_2025_62806_MOESM5_ESM.pdf]

nature portfolio

Corresponding author(s): Yuki Toyama, Koh Takeuchi, Ichio Shimada

Last updated by author(s): Jul 29, 2025

Reporting Summary

Nature Portfolio wishes to improve the reproducibility of the work that we publish. This form provides structure for consistency and transparency in reporting. For further information on Nature Portfolio policies, see our Editorial Policies and the Editorial Policy Checklist.

Statistics

For all statistical analyses, confirm that the following items are present in the figure legend, table legend, main text, or Methods section.

n/a	Confirmed	
☐	☒	The exact sample size (n) for each experimental group/condition, given as a discrete number and unit of measurement
☐	☒	A statement on whether measurements were taken from distinct samples or whether the same sample was measured repeatedly
☐	☒	The statistical test(s) used AND whether they are one- or two-sided<br>*Only common tests should be described solely by name; describe more complex techniques in the Methods section.*
☒	☐	A description of all covariates tested
☒	☐	A description of any assumptions or corrections, such as tests of normality and adjustment for multiple comparisons
☐	☒	A full description of the statistical parameters including central tendency (e.g. means) or other basic estimates (e.g. regression coefficient) AND variation (e.g. standard deviation) or associated estimates of uncertainty (e.g. confidence intervals)
☐	☒	For null hypothesis testing, the test statistic (e.g. F, t, r) with confidence intervals, effect sizes, degrees of freedom and P value noted<br>*Give P values as exact values whenever suitable.*
☒	☐	For Bayesian analysis, information on the choice of priors and Markov chain Monte Carlo settings
☒	☐	For hierarchical and complex designs, identification of the appropriate level for tests and full reporting of outcomes
☒	☐	Estimates of effect sizes (e.g. Cohen's d, Pearson's r), indicating how they were calculated

Our web collection on statistics for biologists contains articles on many of the points above.

Software and code

Policy information about availability of computer code

Data collection	Bruker TopSpin version 3.5pl7, 4.1.4, or 4.2.0 for NMR data collection. Microcal PEAQ-ITC collection 1.41 for ITC data collection.
Data analysis	NMRPipe 11.1 for NMR data processing. NMRFAM-SPARKY 1.470 for NMR data analyses. CS Analyzer 4 2.4.5 and ImageLab version 6.1.0 for gel image analyses. rG4 detector (https://github.com/OrensteinLab/rG4detector), G4Hunter (https://github.com/AnimaTardeb/G4Hunter), pqsfinder version 2.18.0 for GQ propensity calculations. MacroMoleculeBuilder version 3.4 and UCSF ChimeraX version 1.6.1 for structural modeling. The R and Python scripts used in this study along with the relevant data are available on https://doi.org/10.6084/m9.figshare.27134787.v1.

For manuscripts utilizing custom algorithms or software that are central to the research but not yet described in published literature, software must be made available to editors and reviewers. We strongly encourage code deposition in a community repository (e.g. GitHub). See the Nature Portfolio guidelines for submitting code & software for further information.

Data

Policy information about availability of data

All manuscripts must include a data availability statement. This statement should provide the following information, where applicable:

- Accession codes, unique identifiers, or web links for publicly available datasets
- A description of any restrictions on data availability
- For clinical datasets or third party data, please ensure that the statement adheres to our policy

NMR assignments for the N-IDR of DDX3X have been deposited in the BMRB database under accession numbers 52738 [https://dx.doi.org/10.13018/BMR52738] for

the wild type, 52739 [https://dx.doi.org/10.13018/BMR52739] for the RtoK variant, 52740 [https://dx.doi.org/10.13018/BMR52740] for the FYtoA variant, 52741 [https://dx.doi.org/10.13018/BMR52741] for the RKtoA-1 variant, 52742 [https://dx.doi.org/10.13018/BMR52742] for the RKtoA-2 variant, and 52743 [https://dx.doi.org/10.13018/BMR52743] for the RKtoA-3 variant. Structure data used in this study are available in the Protein Data Bank under accession codes 1RNA [http://doi.org/10.2210/pdb1RNA/pdb]121, 2DB3 [http://doi.org/10.2210/pdb2DB3/pdb]32, 2KBP [http://doi.org/10.2210/pdb2KBP/pdb]45, 2KOC [http://doi.org/10.2210/pdb2KOC/pdb]44, 3IBK [http://doi.org/10.2210/pdb3IBK/pdb]46, 5E7M [http://doi.org/10.2210/pdb5E7M/pdb]26, and 7SXP [http://doi.org/10.2210/pdb7SXP/pdb]48. The protein sequence used in this study is available from UniProt with the accession code of O00571 [https://www.uniprot.org/uniprotkb/O00571/entry] (DDX3X). The transcript sequences used in this study are available from Ensembl with the accession IDs ENST00000356142 (RAC1), ENST00000234111 (ODC1), and ENST00000314557 (MITF). Source data are provided with this paper.

Research involving human participants, their data, or biological material

Policy information about studies with human participants or human data. See also policy information about sex, gender (identity/presentation), and sexual orientation and race, ethnicity and racism.

Reporting on sex and gender	N/A
Reporting on race, ethnicity, or other socially relevant groupings	N/A
Population characteristics	N/A
Recruitment	N/A
Ethics oversight	N/A

Note that full information on the approval of the study protocol must also be provided in the manuscript.

Field-specific reporting

Please select the one below that is the best fit for your research. If you are not sure, read the appropriate sections before making your selection.

☒ Life sciences ☐ Behavioural & social sciences ☐ Ecological, evolutionary & environmental sciences

For a reference copy of the document with all sections, see nature.com/documents/nr-reporting-summary-flat.pdf

Life sciences study design

All studies must disclose on these points even when the disclosure is negative.

Sample size	No statistical methods were used to predetermine sample size. For all data sets provided in this work, we chose replicate size according to standard methods provided in the literature and to our own preliminary experiments so as to provide sufficient power for statistical comparison (where applicable).
Data exclusions	The data were not excluded from the analyses.
Replication	All attempts at replication were successful. The numbers of experiments are described in the Methods section or figure legend, where applicable.
Randomization	Since no experimental groups were assigned and all experimental parameters were precisely controlled in the in vitro experiments, randomization was not applicable to this study.
Blinding	Blinding is not relevant for this study because no group allocation was performed.

Reporting for specific materials, systems and methods

We require information from authors about some types of materials, experimental systems and methods used in many studies. Here, indicate whether each material, system or method listed is relevant to your study. If you are not sure if a list item applies to your research, read the appropriate section before selecting a response.

Materials & experimental systems

n/a	Involved in the study
☒	☐ Antibodies
☒	☐ Eukaryotic cell lines
☒	☐ Palaeontology and archaeology
☒	☐ Animals and other organisms
☒	☐ Clinical data
☒	☐ Dual use research of concern
☒	☐ Plants

Methods

n/a	Involved in the study
☒	☐ ChIP-seq
☒	☐ Flow cytometry
☒	☐ MRI-based neuroimaging

Plants

Seed stocks	N/A
Novel plant genotypes	N/A
Authentication	N/A
